# Species determination using the red blood cells morphometry in domestic animals

**DOI:** 10.14202/vetworld.2016.960-963

**Published:** 2016-09-11

**Authors:** Nezar Adili, Mohamed Melizi, Hadj Belabbas

**Affiliations:** 1 Department of Veterinary Medicine, Institute of Veterinary Sciences and Agricultural Sciences, University of Batna 1, Batna 05000, Algeria; 2 Department of Microbiology and Biochemistry, Faculty of Sciences, University of Mohamed BOUDIAF, M’sila 28000, Algeria

**Keywords:** determination, morphometry, red blood cell, species

## Abstract

**Aim:**

This investigation is placed in the context of continuity of a preliminary study already published; it was conducted in cattle, sheep, goats, horses, and dogs; the main aim is to reveal and develop criteria for the animal species determination based on the morphometric parameters of red blood cells.

**Materials and Methods:**

Blood samples were taken from the jugular vein; and the smears were confectioned on slides immediately after the blood collection and stained according to the May-Gründwald Giemsa method. For the morphometric study, three parameters were considered which are: The diameter, the circumference, and the surface of erythrocytes; and measurements were achieved using the OPTIKATM Vision Pro software. Statistical analysis was performed by both analysis of variances and Student’s t analytical tests.

**Results:**

The recorded data showed that the three morphometric parameters of red blood cells are higher in dogs followed, respectively, by those of horses, cattle, and sheep, whereas, the goats have the lowest ones. In addition, the obtained results allowed us to propose new reference values for the circumference and the surface of erythrocyte in considered species.

**Conclusion:**

This investigation permit concluding that from a drop of blood it is possible to characterize the different animal species, taking into account the diameter, the circumference, and the surface of erythrocytes.

## Introduction

During several and even until the last years, morphometric studies of red blood cells, which have been the subject of several studies in various animal species, are essentially based on the linear measurements of erythrocytes size. The only valid and recognized method for measuring the diameter of erythrocytes requires the use of an ocular micrometer and an objective micrometer. The diameter of the red blood cells is measured or estimated roughly under an optical microscope at a magnification of immersion (×100) [1,2].

The cattle red blood cells have a width of 5-6 μm, with mild to moderate physiologically anisocytosis [3]. Sheep erythrocytes have a diameter of 4-5 μm; however, red blood cells of goats are the smallest compared to other species with 2.5-3.9 μm of width [4]. The equine red blood cell measure 5-6 μm in diameter with physiologically common rouleaux formation showed in blood smears [5-7]. The dog’s erythrocytes are larger compared to other species; their diameter varies from 6 to 8 μm [8].

Further, morphometric studies of red blood cells showed several morphological changes; the most important is due to age and breed. Fetal red blood cells are larger than those of adult animals; the diameter of the erythrocyte continues to decrease during the first 9-12 weeks of life due to the replacement of fetal red blood cells by the adult erythrocytes [9,10]. In horses, the red blood cells of both Arabian and English thoroughbred are smaller than those in common breeds [11,12]. In dogs, poodles have a constitutional macrocytosis; conversely, some Japanese breeds (Akita and Sheba) have naturally small red cells [8,9].

This work is the continuity of the preliminary study realized only in females, which allowed us to seem very highly significant differences in the morphometry of erythrocytes between species [13]. The current study was performed in males and females of several breeds of cattle, sheep, goats, horses, and dogs; it aims to complete and confirm the results already published and also to define and develop criteria selection of domestic animals species, by considering three morphometric parameters of red blood cells which are the diameter, the circumference, and the surface.

## Materials and Methods

### Ethical approval

All samples were collected as per the standard blood collection method without any stress/harm to animals. The committee framed for the research by the university authority approved the investigation. During the study visits, the researchers introduced themselves and explained the objective and methodology of the study to all animal breeders.

### Animals

This study was undertaken in the east of Algeria, and it was carried out on cattle, sheep, goats, horses, and dogs belonging to the following breeds: The local small Brown of Atlas and the crossing breed (mostly from local and imported breeds) for cattle; the two local Ouled-Djellal and Hamra breeds for sheep; the Arbia local goat; the Arabian and English thoroughbred for horses; the local Sloughi and the German Shepherd in Dogs. For each breed, 30 subjects were selected; clinically healthy animals, free from internal and external parasites were divided according to their sex in two batches as follows: 15 adult males and 15 non-pregnant adult females.

### Blood samples and smears

All blood samples were taken by puncture of the jugular vein; and smears were confectioned on microscope slides directly after venipuncture without the use of anticoagulants, which can induce some changes in the morphology and the morphometry of blood cells [14-16].

### Blood smears staining

Staining of blood smears was conducted following the Romanowsky staining method, especially by the dye of May-Gründwald Giemsa the most appropriate staining to mark red blood cells of mammals, respecting always the protocol cited by Houwen [17], Harvey [18], and Ledieu [19].

### Morphometric study of red blood cells

The study of the morphometric parameters of red blood cells was performed with special OPTIKA™ Vision Pro software, of the optical microscope OPTIKA B-350 [13]. Initially, the diameter of red blood cells was measured, which is still estimated in relation to the shape of the cell; then, two new parameters that have not previously treated were developed, which are the circumference and the surface of erythrocytes. The morphometric study of red blood cells was performed by always respecting the guidelines and instructions of the software manufacturer. For each animal, the diameter, the circumference, and the surface of 50 red blood cells were measured, with determination of their averages and this for all the species and breeds.

### Statistical study and analysis of results

To better assess the statistical significance of the experimental data and the influence of the diameter, the circumference, and the surface of erythrocytes on the determination of the species, both analysis of variances and Student’s t-test were performed with the MedCalc statistical software (Version 12.7, Copyright © 1993-2013 MedClac software bvba). Values obtained were expressed as means with standard deviations; the statistical signification was set at p under 0.05 (5%).

## Results

To study more precisely the influence of the three morphometric parameters of red blood cell (diameter, circumference, and surface) on the determination of pets, species and breeds considered in this study were randomized into two groups, as follows:

- First group: We compare the obtained results between local Brown of Atlas Cattle, Ouled-Djellal sheep, Arbia local goat, the Arabian thoroughbred horse, and the local dog
- Second group: We compared the values observed in: Crossing cattle, local Hamra sheep, Arbia goat, the English thoroughbred horse, and German shepherd dog.

### Study of the observed results in the first group

Results indicated in Table-1 show the influence of the diameter of erythrocytes on the determination of the species, where they are very significantly larger in local dogs followed, respectively, by those of Arabian horses, local cattle, Ouled-Djellal sheep, and finally by the group of Arbia goats (with always p<0.001).

**Table-1:** Influence of red blood cells diameter on the species determination of pets (Group 1, expressed in μm).

Groups	Brown of Atlas cattle	Ouled-Djellal sheep	Arbia goats	Arabian horses	Local dogs
Global (n=30)	5.12±0.20^a^	4.46±0.19^b^	3.39±0.12^c^	5.56±0.27^d^	7.12±0.28^e^
Adult males (n=15)	5.00±0.22^a^	4.36±0.16^b^	3.38±0.11^c^	5.52±0.32^d^	7.23±0.23^e^
Adult females (n=15)	5.23±0.10^a^	4.55±0.17^b^	3.40±0.13^c^	5.59±0.21^d^	7.01±0.30^e^

a,b,c,d,e Mean values in the same rows with different superscripts letters are significantly different (p<0.001)

Results regarding the influence of the circumference of red blood cells on the determination of the species in domestic animals are presented in Table-2. After comparing the averages, it appears that there are very highly significant differences between species (p<0.001) with always an advantage for dogs against other species.

**Table-2:** Influence of red blood cells circumference on the species determination of pets (Group 1, expressed in μm).

Groups	Brown of Atlas cattle	Ouled-Djellal sheep	Arbia goats	Arabian horses	Local dogs
Global (n=30)	19.40±1.51^a^	16.93±0.68^b^	12.94±0.72^c^	22.77±1.73^d^	25.72±1.50^e^
Adult males (n=15)	18.15±1.06^a^	16.56±0.61^b^	12.58±0.62^c^	23.41±1.97^d^	25.57±0.96^e^
Adult females (n=15)	20.65±0.55^a^	17.31±0.53^b^	13.29±0.65^c^	22.13±1.19^d^	25.88±1.92^e^

a,b,c,d,e Mean values in the same rows with different superscripts letters are significantly different (p<0.001)

Changes relative to the influence of the surface of red blood cells on the determination of the species are shown in Table-3. As with the both first parameters, we note that the erythrocyte surface presents also the greatest values in dogs, whereas the lowest values are recorded in goats; it should be noted that all the differences are statistically very highly significant (p<0.001).

**Table-3:** Influence of red blood cells surface on the species determination of pets (Group 1, expressed in μm^2^).

Groups	Brown of Atlas cattle	Ouled-Djellal sheep	Arbia goats	Arabian horses	Local dogs
Global (n=30)	22.56±2.49^a^	16.38±1.26^b^	9.50±0,96^c^	27.93±3.24^d^	38.83±4.30^e^
Adult males (n=15)	20.66±1.83^a^	15.69±1.03^b^	9.15±0,96^c^	28.39±3.71^d^	38.08±2.90^e^
Adult females (n=15)	24.47±1.30^a^	17.80±1.09^b^	9.84±0,86^c^	27.47±2.74^d^	39.57±5.35^e^

a,b,c,d,e Mean values in the same rows with different superscripts letters are significantly different (p<0.001)

### Study of the observed results in the second group

From the observation of data in Table-4, it appears that the differences are very marked between all groups (p<0.001), the highest value is recorded in German shepherd dog, and then successively in purebred English horses, mixed cattle, and the Hamra sheep, whereas local goats have the lowest value.

**Table-4:** Influence of red blood cells diameter on the species determination of pets (Group 2, expressed in μm).

Groups	Crossing cattle	Hamra sheep	Arbia goats	English horses	German Shepherd
Global (n=30)	5.02±0.14^a^	4.38±0.13^b^	3.90±0.12^c^	5.76±0.18^d^	6.92±0.24^e^
Adult males (n=15)	4.98±0.12^a^	4.39±0.15^b^	3.38±0.11^c^	5.76±0.19^d^	6.95±0.23^e^
Adult females (n=15)	5.05±0.16^a^	4.37±0.12^b^	3.40±0.13^c^	5.75±0.18^d^	6.88±0.26^e^

a,b,c,d,e Mean values in the same rows with different superscripts letters are significantly different (p<0.001)

Table-5 also shows that the circumference of red blood cells of the German dog’s group is clearly the most superior, followed again by those of English thoroughbred horses, crossing cattle, Hamra sheep, and finally by the group of Arbia goats.

**Table-5:** Influence of red blood cells circumference on the species determination of pets (Group 2, expressed in μm).

Groups	Crossing cattle	Hamra sheep	Arbia goats	English horses	German Shepherd
Global (n=30)	18.57±0.69^a^	16.89±0.72^b^	12.94±0.72^c^	22.14±0.84^d^	25.61±0.95^e^
Adult males (n=15)	18.19±0.39^a^	16.70±0.59^b^	12.58±0.62^c^	22.54±0.72^d^	25.89±0.85^e^
Adult females (n=15)	18.95±0.72^a^	17.08±0.81^b^	13.29±0.65^c^	21.74±0.78^d^	25.33±0.99^e^

a,b,c,d,e Mean values in the same rows with different superscripts letters are significantly different (p<0.001)

The distribution of the different groups registered in Table-6, noted a very marked effect of the erythrocytes surface (p<0.001); this parameter shows very high values for dogs, then horses, cattle, and sheep, respectively, whereas the very low values are those of goats.

**Table-6:** Influence of red blood cells surface on the species determination of pets (Group 2, expressed in μm^2^).

Groups	Crossing cattle	Hamra sheep	Arbia goats	English horses	German Shepherd
Global (n=30)	20.45±1.30^a^	16.49±0.97^b^	9.50±0.96c	27.47±2.05^c^	38.51±3.05^e^
Adult males (n=15)	20.34±0.98^a^	16.50±1.16^b^	9.15±0.96c	28.29±1.81^c^	39.04±2.98^e^
Adult females (n=15)	20.56±1.58^a^	16.8±0.77^b^	9.84±0.86c	26.65±2.00^c^	37.98±3.14^e^

a,b,c,d,e Mean values in the same rows with different superscripts letters are significantly different (p<0.001)

## Discussion

The morphometric study of red blood cell seems to show that the diameter, the circumference, and surface of erythrocytes have a significant influence on the determination of the species of domestic animals. For the diameter of erythrocytes in different species, it is clear that the obtained values are similar and always correspond to international reference values cited by the authors in the previous study [3,4,7,8]; the size of red blood cells is greater in dogs followed by the horses, cattle, and sheep, respectively, whereas the goats have smaller red blood cells.

Regarding the circumference and the surface of erythrocytes, the recorded results also show that these two parameters are also higher in dogs while the lowest values are observed in goats (Table-7); these results point in the same direction and the same trend as the results obtained in the preliminary study [13].

**Table-7:** Summary of new reference values of morphometric parameters of red blood cells in pets.

Species	Diameter (in μm)		Circumference (in μm)		Surface (in μm^2^)	
		
	Mean	Range	Mean	Range	Mean	Range
Bovines	5.07	4.66-5.50	18.98	17.28-20.25	21.51	18.57-26.50
Ovines	4.42	4.10-4.62	16.91	15.73-19.72	16.44	14.37-19.03
Goats	3.39	3.09-3.60	12.94	11.60-14.65	9.50	7.61-11.28
Horses	5.66	4.72-6.07	22.46	19.01-26.99	27.70	19.38-34.32
Dogs	7.02	6.30-7.71	25.67	20.63-28.31	38.67	25.55-47.05

This investigation allowed us to note that the traditional method of red blood cells diameter measuring, based on the use of an ocular micrometer and a microscopic slide, is an estimate technique, imprecise, and quite difficult.

Measurements of the circumference and the surface of erythrocytes using the evoked software perform much more precise morphometric studies, limiting the human factor, which is involved in the studies with ocular micrometer by choosing the best place. It appears that the morphometric study of red blood cells based on the use of software-like OPTIKA™ Vision Pro is practical and direct; more this new measurement method is easy to perform, fast, and very inexpensive.

## Conclusion

In the light of the obtained results, it turns out that morphometric studies of erythrocytes performed by sophisticated and advanced software are more appropriate and more precise than conventional measurements with the ocular micrometer. Finally, through this study, we can conclude that from a drop of blood, it is possible to characterize animal species, taking into account the morphometric parameters of red blood cells. Therefore, it would be worthy making new morphometric studies of erythrocytes in other species and breeds, using this type of software.

## Authors’ Contributions

NA and MM conceived, designed the work, wrote and revised the manuscript. Both NA and HB conducted the experiment and analysis. All authors read and approved the final manuscript.
